# Entomopathogenic Fungi in Forest Habitats of *Ixodes ricinus*

**DOI:** 10.3390/insects15050341

**Published:** 2024-05-09

**Authors:** Dagmara Dyczko, Kinga Plewa-Tutaj, Dorota Kiewra

**Affiliations:** Department of Microbial Ecology and Acaroentomology, Faculty of Biological Sciences, University of Wrocław; 51-148 Wrocław, Poland; dagmara.dyczko2@uwr.edu.pl (D.D.); dorota.kiewra@uwr.edu.pl (D.K.)

**Keywords:** entomopathogenic fungi, *Metarhizium*, *Beauveria*, *Ixodes ricinus*

## Abstract

**Simple Summary:**

This study shows the common occurrence of entomopathogenic fungi (EF) in the habitats of *Ixodes ricinus*, which indicates the necessity to consider EF as a potential factor influencing the tick population. However, the obtained results highlight the need for further research to fully understand the interaction between soil microorganisms and tick occurrence.

**Abstract:**

(1) Background: In addition to the microclimate, host availability, and tick microbiota, soil environmental microorganisms can affect tick populations. This study aimed to (1) determine the presence and diversity of entomopathogenic fungi (EF) in forests, where ticks are abundant, and (2) estimate the effectiveness of the isolated EF strains against *Ixodes ricinus*. (2) Methods: EF were isolated using the trap insect method from soil collected from tick sites. A bioassay was used to estimate the effectiveness of EF against ticks. (3) Results: The presence of EF was found in all tested forest habitat types. A total of 53 strains belonging to the genera *Metarhizium*, *Beauveria*, and *Isaria* were isolated. All the six strains subjected to the bioassay showed potential efficacy against both adult and nymphal stages of *I. ricinus*; however, the strains differed in their effectiveness. The most effective isolate against *I. ricinus* was the soil environmental strain of *Metarhizium anisopliae*. (4) Conclusion: The study indicates that tick habitats can be the source of entomopathogenic fungi, which have a lethal effect on ticks, as demonstrated in preliminary laboratory tests with *I. ricinus*. However, for practical use, extensive field tests and further research on application methods and long-term effects are necessary to develop effective and sustainable tick management strategies.

## 1. Introduction

Ticks (Ixodida) are vectors of many pathogens and pose a serious threat to human and animal health. In Europe, *Ixodes ricinus* is the most abundant tick species, with the greatest role in spreading a wide variety of pathogens [1,2,3]. In recent years, research has mainly focused on molecular methods of pathogen detection and characterization, while the basic biology and ecology of *I. ricnus* have been relatively neglected [4]. There is still a knowledge gap related to the factors affecting ticks during the non-parasitic life phase [5]. Meanwhile, understanding the biotic and abiotic factors that influence the tick population is key to developing effective control strategies and reducing the risk of pathogen transmission [6,7]. The low survival rates of the different life stages (about 5% of eggs, 10% of larvae, and 20% of nymphs) indicate the existence of natural mechanisms regulating their populations [8]. *I. ricinus* spends the vast majority of its life, >99%, as a free-living (off-host) organism [4]. During the off-host phase, including the engorged phase after detaching, molting, and the unfed phase, ticks are particularly exposed to external environmental factors, including temperature and saturation deficit. To avoid dehydration, they take shelter in the deep leaf litter close to the soil surface [5]. Factors within the soil ecosystem, like slope aspect, hydrology, and soil texture, influence off-host tick survival. Ticks are also influenced by biotic factors, including plants, insectivorous vertebrates, soil-dwelling arthropod predators, and the soil microbiota [5,9]. In the soil environment, ticks can come into contact with potentially lethal entomopathogenic fungi (EF) spores because forest soils provide a convenient habitat for EF [10]. Entomopathogenic fungi play a crucial role in limiting arthropod populations. They can be used to suppress arthropods, including insects and, as mycoacaricides, mites and ticks of economic, medical, and veterinary importance [11]. Moreover, several tick species are naturally infected by EF [12,13].

The lethal effects of EF vary depending on the developmental stage of ticks, and EF have shown the ability to control these vectors under both laboratory and field conditions, but the performance of these fungi as control agents is highly strain specific and requires further research [14]. Furthermore, there is a notable degree of genetic variability within a single entomopathogenic species between isolates from disparate geographical locations [15,16,17]. Entomopathogenic fungi produce enzymes, such as proteases, chitinases, and lipases, that degrade host cuticle constituents (proteins, chitin, and lipids), thereby facilitating hyphal penetration. Furthermore, some EF species are capable of producing toxic compounds, including beauvericin, destruxin, bassianolide terpenes (trichocaranes and fumosorinone), lactone compounds (cepharosporolides), acids (dipicolinic acid and oxalic acid), and others [18,19,20,21]. However, not all fungal species have the same ability to produce enzymes or toxins in terms of diversity, quantity, and activity. EF are considered to be pathogens that are non-specific and can infect both mites and insects. However, they have not been shown to develop specificity against ticks [22]. Mortality rates are also generally higher in laboratory studies than in the field [23]. It is worth emphasizing that locally isolated entomopathogenic fungi are better adapted to the natural conditions of their geographical locations [24]. Therefore, it is important to obtain new, locally occurring isolates, particularly from tick sites. These isolates are essential in developing effective biocontrol agents for the native species of these arthropods. It is important to note that research has demonstrated that certain strains of fungi, including *Metarhizium anisopliae* and *Beauveria bassiana*, may display alterations in virulence following prolonged laboratory cultivation. It has been proposed that these changes could potentially be mitigated by periodic re-isolation of the fungi from the field or, alternatively, by using preservation techniques that minimize genetic alterations [24].

The efficacy of entomopathogenic fungi under laboratory conditions against various tick species and life stages and in reducing the number of eggs laid by infected females has been found in many studies [22,25,26,27,28,29,30]. Many studies have also found that entomopathogenic fungi can effectively reduce the abundance of ticks in the wild. The most extensively studied entomopathogenic fungi for the biological control of tick abundance are *Metarhizium anisopliae* and *Beauveria bassiana* [31]. However, research on EF efficacy mainly focuses on tick species that are not found in Europe, such as *Ixodes scapularis*, *Rhipicephalus microplus*, *Amblyomma americanum*, and *Dermacentor variabilis* [27,32,33]. Data assessing the efficacy of entomopathogenic fungi against the most important tick species in Europe, particularly *Ixodes ricinus* and *Dermacentor reticulatus*, are limited [34,35,36,37]. Previous studies on the effects of EF on *I. ricinus* have mainly focused on the larvae and nymphs of this species [34,35], engorged females [36], and unfed adults [37]. Moreover, research on EF focuses mainly on identifying potential biological control agents and less on the natural distribution of EF in soils and interactions with ticks [5].

Therefore, the aim of the study was to (1) determine the presence and diversity of entomopathogenic fungi (EF) in forests, where ticks are abundant, and (2) estimate the efficacy of EF isolates against *Ixodes ricinus*.

## 2. Materials and Methods

### 2.1. Study Area

Field surveys were conducted in three forest habitat types classified in Poland: broadleaf forest (BF), mixed broadleaf and coniferous forest (MBCF), and coniferous forest (CF). The forest habitat type was determined using land cover maps available in the Forest Data Bank (https://www.bdl.lasy.gov.pl/portal/mapy, accessed on 24 April 2019). Finally, in the Miękinia Forest District of Lower Silesia, SW Poland, 9 sites (3 sites in each forest habitat type) harboring *I. ricinus* [38] were selected for the study. All sites were located within the forest complex and, in some cases, close to each other, within a few hundred meters, to avoid ecotone effects. The detailed characteristics of the sites have been described by Dyczko et al. [38].

### 2.2. Soil Sampling

To isolate entomopathogenic fungi, soil samples were taken from the tick sites. From each site, four bulk soil samples weighing approximately 1 kg were collected from a depth of up to 15 cm. Each bulk sample was a mixture of five primary samples taken from evenly spaced points, including four corners of the field and one from the center. Each soil sample was collected using a disinfected metal shovel to prevent cross-contamination. The soil samples were placed in individual plastic buckets and transported to the laboratory. They were then sieved through a 2 mm sieve to remove larger roots and stones. The prepared soil samples were stored at 4 °C for up to 4 weeks before further microbiological testing according to Pérez-González et al. (2014) [39]. A total of 72 soil samples were collected from nine locations during the 2018 and 2019 seasons.

### 2.3. Isolation of Entomopathogenic Fungi

**Figure 1:**
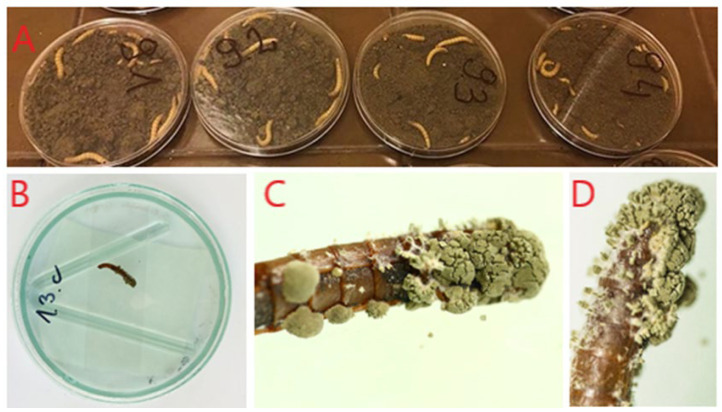
Trap insect method (original): (**A**) soil samples containing *T. molitor* larvae, (**B**) humid chamber containing *T. molitor* larvae, and (**C**,**D**) infected *T. molitor* larvae (with fungal spores produced).

Entomopathogenic fungi were isolated from the soil samples collected using the trap insect method (Zimmermann, 1986) with larvae mealworm (*Tenebrio molitor*) (Figure 1) [40]. The procedure involved placing ten *T. molitor* larvae on 80g of the tested soil sample, which was then moistened with sterile distilled water and placed on sterile Petri dishes. The dishes were stored in the dark at 20–22 °C. During the initial 3 days, the plates were inverted every 24 h to encourage larval movement within the soil and enhance the probability of contact with fungal spores. If required, sterile distilled water was used to moisten the soil. The evaluation of larval infection by entomopathogenic fungi was conducted every 3 days for a period of 2 weeks or until all larvae had perished [41]. Dead, infected larvae were transferred and incubated in humid chambers, which were sealed sterile Petri dishes lined with sterile moist filter paper. This was done to increase mycelial growth and sporulation on the insect body surface. The mycelium from the insect cuticle was then stab-inoculated using a sterile dissecting needle on SAB (Sabouraud) medium with chloramphenicol. This medium inhibits the growth of bacteria present in the tested sample, allowing for fungal growth. To obtain pure sporulating colonies, isolates were inoculated onto potato dextrose agar (PDA) medium. The fungal isolates were stored on slants with an appropriate medium under refrigerated conditions. They were maintained with a transfer series. The fungi were initially identified to the genus using a key for determining entomopathogenic fungi [42] based on the observation of morphological features, including macro- and microscopic images, using a light microscope. Preparations for microscopic observation were stained with lactophenol (Sigma, Burlington, MA, USA).

### 2.4. DNA Extraction

Fungal conidia were obtained using the trap insect method from one or two infected *T. molitor* larvae from each soil sample. DNA was extracted from pure sporulating colonies. DNA extraction was performed according to the methodology described by Kepler et al. [43]. Conidia were inoculated into small Petri dishes (3 cm diameter) containing PDA medium. The Petri dishes were then incubated for 7 to 10 days without access to light at 23 ± 2 °C. After the incubation period, the conidia were scraped from the medium using a scalpel blade and then transferred to 2 mL microtubes (in accordance with the isolation instructions provided in the commercial kits used). The resulting supernatant was transferred to a sterile tube and stored at −20 °C until further analysis. The extracted DNA was used as a template for polymerase chain reaction (PCR).

### 2.5. Molecular Identification of Entomopathogenic Fungi

The primer pair ITS4 (5′-TCCTCCGCTTATTGATATGC-3′) and ITS5 (5′-GGAAGTAAAAGTCGTAACAAGG-3′) [39] was used to amplify the fungal internal transcribed spacer (ITS). The reaction mixture for a single sample had a volume of 25 μL: 12.5 μL 2×PCR Mix Plus (A&A Biotechnology, Gdansk, Poland), 1.25 μL of each primer, 7 μL of sterile nuclease-free water, and 3 μL of template DNA. PCR conditions included initial denaturation at 95 °C for 3 min. The protocol for PCR amplification involved 35 cycles of denaturation at 95 °C for 30 s, primer annealing at 45 °C for 1 min, extension at 72 °C for 1 min, and a final extension at 72 °C for 5 min.

PCR products were separated on 1.5% agarose gel with the addition of SimplySafe (Eurx, Gdańsk, Poland). The results of the electrophoretic separation were visualized under UV light and stored on computer memory using Quantity One Basic (Bio-Rad, Hercules, CA, USA). A positive reaction was defined as the presence of a product of approximately 600 bp [39] The DNA of positive samples was purified using a DNA purification kit (GenoPlast Biochemicals, Rokocin, Poland) according to the manufacturer’s protocol, and nucleotide sequences were determined by a specialized company (Genomed, Warsaw, Poland). Obtained nucleotide sequences were compared with sequences available from the National Center for Biotechnology Information (NCBI) https://blast.ncbi.nlm.nih.gov/Blast.cgi (accessed on 13 March 2023). The sequences have been deposited in the GenBank database and can be accessed via the following accession numbers: PP713035-PP713040.

### 2.6. Tick Collection for Bioassays

Tick specimens used in the bioassay were collected from vegetation in forest areas in Lower Silesia during the peak spring activity period of *I. ricinus* in both 2018 and 2019. Ticks were collected on dry and windless days between 9:00 and 15:00 using standard flagging methods and placed in plastic containers with a green leaf to maintain proper humidity conditions. The collected specimens were kept refrigerated at 6 ± 2 °C until the bioassay was performed, with a maximum storage time of 14 days. A total of 2160 ticks identified as *I. ricinus* were used, comprising 720 females, 720 males, and 720 nymphs.

### 2.7. Sporulation Test and Bioassays

Biological tests were conducted under laboratory conditions to assess the efficiency of isolated entomopathogenic fungi against *I. ricinus*. Before the bioassay, all fungal strains were cultured on PDA medium in standard 90 mm Petri dishes at 22 °C for 3 weeks [29,44]. The mature colonies were then scraped into 0.1% Tween 80. The entire pellet was centrifuged at 4000 rpm for five minutes to separate the spores (upper fraction) from the filaments. This was followed by a series of dilutions of the resulting suspensions (10^−^^1^, 10^−^^2^, 10^−^^3^). The concentration of spores from the diluted sample (10^−^^2^ or 10^−^^3^) was measured in a Fuchs–Rosenthal chamber.

Before conducting the bioassay, a spore germination test was carried out. The prepared suspension (1 mL) was incubated in PDA medium at room temperature for 18 h. The ratio of germinating to non-germinating spores (%s) was determined using the formula: %s = (gs/ngs) × 100 (%), where gs represents the number of germinating spores and ngs represents the number of non-germinating spores. Only strains with a germination rate of over 90% were used in the bioassay.

The lethality of each strain was tested by immersing an *I. ricinus* adult or nymph in a suspension of spores [44,45]. To remove any external contaminants from the ticks, they were washed with a sterile isotonic solution supplemented with 0.9% sodium chloride. They were then immersed in the spore suspension for three minutes. For each developmental stage of the ticks, initial concentrations and their 10- and 100-fold dilutions were used for the bioassay. To obtain repeatable results for each strain and dilution, 3 replicates were performed, and a control group, which was immersed in a sterile aqueous solution of 0.1% Tween 80, was also used. After immersion in the spore suspension, the ticks were transferred to sterile containers containing moist filter paper and incubated for 21 days. The ticks were kept at 23 ± 1 °C and 80% relative humidity in the absence of light. Mortality was observed daily for a period of 3 weeks. Tick paralysis, which is characterized by erect legs and a lack of response to stimuli, such as heat, light, and CO_2_, as well as traces of mycelial overgrowth on the body surface, were considered lethal effects. Dead specimens were separated and incubated in humid chambers to stimulate mycelial growth on the cuticle surface.

### 2.8. Statistical Analyses

The impact of entomopathogenic fungi on ticks was measured by determining the dose required to cause 50% mortality (LC_50_). LC_50_ values were calculated using Finney’s (1952) probit analysis method with the LC_50_/LD_50_ calculator, which is specifically designed to calculate dosages with Abbot’s correction [46].

## 3. Results

**Figure 2:**
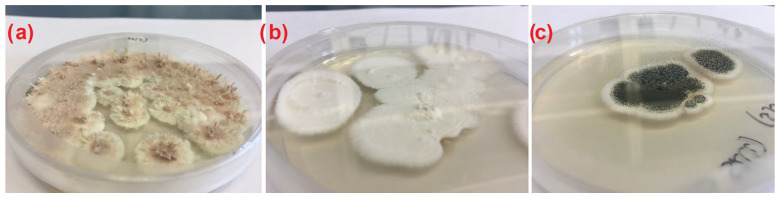
Macromorphology of (**a**) *Isaria* spp., (**b**) *Beauveria* spp., and (**c**) *Metarhizium* spp. on PDA medium (original).

The entomopathogenic fungi were isolated from 7 of 9 sites in three forest habitat types (broadleaf forest, mixed broadleaf and coniferous forest, and coniferous forest (Table 1)). In total, 53 isolates were obtained from 72 soil samples. Macro- and microscopic observations allowed the identification of EF species belonging to the genera *Metarhizium*, *Beauveria*, and *Isaria* (Table 2 and Figure 2). Detailed sequence analysis of 13 strains randomly selected from each site confirmed genera determined microscopically and additionally allowed identification of 8 isolates to the species level.

The number of isolates and species varied among sites and forest habitat types. The commonest EF in the soil was *Metarhizium* (37 isolates, 69.8%), followed by *Isaria* (11 isolates, 20.8%) and *Beauveria* (5 isolates, 9.4%). The largest number of isolates of EF and the greatest diversity were obtained from broadleaf forests (BFs). Strains obtained from this forest habitat type accounted for 66.0% of all isolates, including 20 strains of *Metarhizium*, 4 of *Beauveria*, and 11 of *Isaria*. The study showed that 24.5% (13 strains) of entomopathogenic fungi strains were isolated from mixed broadleaf and coniferous forests (MBCFs), including 12 *Metarhizium* and 1 *Beauveria* strains. From coniferous forests (CFs), only five strains of *Metarhizium* were isolated, accounting for 9.5% of the total strains.

**Figure 3:**
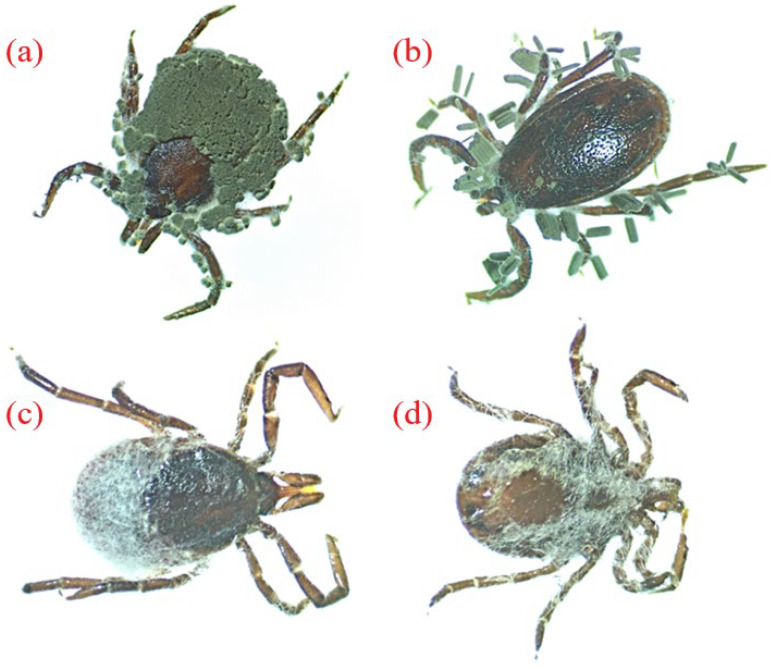
*I. ricinus* specimens (**a**) female and (**b**) male infected with *Metarhizium* sp. and (**c**) female and (**d**) male infected with *Beauveria* sp. (original).

The bioassay covered six environmental isolates, including four strains of *Metarhizium* and two strains of *Beauveria* (Table 2, Figure 3). All the six strains subjected to the bioassay showed a potential lethal effect against both adult and nymphal stages of *I. ricinus;* however, the strains differed in their effectiveness. The efficacy of the entomopathogenic fungi was assessed on the basis of the percentage of tick mortality required to calculate the median lethal concentration (LC_50_). The most effective EF isolates against *I. ricinus* were two strains of *Metarhizium anisopliae*: 3.4(2) and 6.4(6). Strain 3.4(2) was collected from the soil in CFs, while strain 6.4(6) from was collected from the soil in BFs. The LC_50_ for strain 3.4(2) *M. anisopliae* ranged from 1.6 × 10^5^ cfu/mL for males up to 1.4 × 10^6^ cfu/mL for nymphs, while that for strain 6.4(6) *M. anisopliae* ranged from 2.9 × 10^5^ cfu/mL for females up to 1.9 × 10^6^ cfu/mL for nymphs. For most strains, female ticks were more sensitive to *Metarhizium* compared to males and nymphs. *Beauveria* strains were less effective: the LC_50_ value for nymphs ranged from 2.6 × 10^6^ cfu/mL (strain 1.4(4)) to 6.6 × 10^7^ cfu/mL (strain 2.3(1)).

## 4. Discussion

Our study showed that entomopathogenic fungi (EF) are widespread in the soil collected from tick habitats, because EF were isolated from the soil collected from all tested types of forest where *I. ricinus* are abundant. However, the number of isolates and species of EF varied among sites and forest habitat types. Among the forest habitats selected, which according to the Forest Data Bank www.bdl.lasy.gov.pl (accessed on 24 April 2019) represent the largest area of forest habitats in Poland, the largest number of isolates of EF and the greatest diversity of EF were obtained from broadleaf forests (BFs). Strains obtained from BFs accounted for 66.0% of all isolates and included the EF of three genera: *Metarhizium*, *Beauveria*, and *Isaria*. Fewer EF isolates were obtained from the soil collected from mixed broadleaf and coniferous forests (MBCFs) and coniferous forests (CFs). In this study, we did not statistically confirm the relationship between the occurrence of EF and tick abundance due to the insufficient sample size, which restricts the statistical power needed to detect potentially ecological interactions; however, the impact of EF on local tick populations cannot be excluded. Future studies should aim to collect more extensive data across different geographical regions, including broadleaf forests (BFs), where the largest number of EF was isolated and, as previous studies have shown [38], the largest number of ticks was obtained, to enhance the robustness of statistical analyses. The observed higher number and diversity of EF isolates, such as *Metarhizium*, *Beauveria*, and *Isaria*, in broadleaf forests (BFs) could be influenced by interactions with both abiotic and biotic factors, including soil pH, soil type, method of soil cultivation, organic matter content, temperature, humidity, and host density [47,48,49]. The influence of other microorganisms cannot be excluded either; especially, mycorrhizal fungal communities, which form symbiotic relationships with the roots of most plant species, play crucial roles in nutrient cycling, soil structure, and maintaining moisture levels [50,51,52].

The important role of soil in forest areas in maintaining EF is also documented in other studies. Popowska-Nowak et al. (2016) conducted studies in various regions of Poland [53] and found that *M. anisopliae* and *I. fumosorosea* are the most commonly isolated EF species from the soil of several-year-old forest nurseries, particularly in spring. Majchrowska-Safaryan and Tkaczuk (2021) found in coniferous, deciduous, and mixed forests the presence of *Beauveria* spp., *Cordyceps* spp., *Metarhizium* spp., and *Lecanicillium* spp.; however, the mean densities of EF varied by forest type, sampling date, and soil depth [54]. In addition, they found that the densities of EF were usually higher in leaf litter than in soil; however, Tuininga et al. (2009), taking into account EF from soil, leaf litter, and ticks in a forested area known to harbor *I. scapularis*, suggested that ticks are more likely to encounter EF in soil than in leaf litter [9].

Differences in the qualitative and quantitative results of the isolated strains may have arisen due to various factors that influence the EF species’ composition and abundance in soil. Furthermore, understanding the composition and distribution of indigenous fungal species is crucial in evaluating their effectiveness and potential use in biological control within a particular ecosystem. Moreover, the rapid pace of environmental change further emphasizes the urgency to expand our knowledge of EF. Understanding how these fungi interact with arthropod hosts in changing ecosystems is crucial for predicting their impacts on tick population dynamics and for developing sustainable tick population management strategies. A sustainable tick population management strategy that leverages entomopathogenic fungi could involve the use of fungi-based biopesticides to control tick populations in a way that is environmentally friendly and reduces reliance on chemical pesticides [33].

Determining the presence of entomopathogens in tick environments is not sufficient to determine their impact on ticks, because different isolates are characterized by different lethality values, and more comprehensive research is required. Bioassays are an important test to verify the virulence of EF isolates and to indicate the most virulent isolates with the greatest potential for biocontrol [12]. We used six environmental isolates in bioassays to evaluate their acaricidal efficacy on ticks, including four strains of *Metarhizium* collected from soil in broadleaf forests (BFs) and coniferous forests (CFs) and two strains of *Beauveria* collected from BFs and mixed broadleaf and coniferous forests (MBCFs). The bioassay results indicated that the potential for tick control is variable among the fungal strains tested; however, all the six environmental fungal isolates that we tested showed potential efficacy against both adult and nymphal stages of *I. ricinus*. Fungi can evolve to increase the success of their biological cycle, which is influenced by the arthropods they use to reproduce [42,55]. We cannot exclude the fact that in the forests, ticks can serve as a host for EF, and therefore, local EF strains may be better adapted to the local tick population. Genomic studies of EF have shed light on the mechanisms that allow their adaptation to different hosts and environments, further supporting their application in pest management. For example, the genera *Beauveria* and *Metarhizium* have been extensively studied for their virulence genes and potential for genetic improvement for enhanced biocontrol capabilities [56,57]. The enhancement of biocontrol capabilities in EF, such as *Beauveria* and *Metarhizium*, can indeed be pursued through both selection pressure cultivation and genetic engineering. Selective breeding and artificial selection involve the selection of fungi with desirable traits and their breeding to produce more effective biocontrol agents. For example, the application of artificial selection for fungicide resistance to *B. bassiana* and *M. brunneum* has demonstrated that selective breeding can enhance specific beneficial traits in these fungi [58]. Furthermore, research has actively used genetic engineering to enhance the virulence and environmental resilience of these fungi. Techniques include the introduction of genes that enhance pathogenicity or resistance to environmental stresses. For instance, a study conducted by Shang et al. (2012) demonstrated that the genetic engineering of *B. bassiana* with a tyrosinase gene is an effective method for improving fungal tolerance against UV damage [59]. Both methods have their respective merits and can be used depending on the research objectives, regulatory considerations, and specific characteristics desired in the fungal strains. While selection pressure relies on naturally occurring genetic diversity and evolution, genetic engineering provides a more rapid and precise method for developing specific traits. Furthermore, EF have clear benefits, including ecological safety, mass production, and the ability to infect their hosts through the cuticle rather than waiting for the host to ingest them to cause infection [60]. While the ecological safety of EF is noted, specific studies on the impact of these fungi on non-target species within the forest ecosystems are crucial. Understanding these interactions is necessary to ensure the ecological integrity of control measures. It has also been reported that EF can affect the entire tick life cycle (free-living and parasitic stages) [61]. The impact of EF on free-living stages (such as larvae, nymphs, and adults in the environment) and parasitic stages (ticks that are attached to hosts) is twofold. This dual impact enables the effective reduction in tick populations. The impact on free-living stages helps to control ticks at multiple stages in their life cycle, potentially reducing the chances of tick survival and reproduction [62]. Furthermore, EF can be applied to the environment to target free-living stages and potentially be used on animal hosts to target parasitic stages [63]. In addition, the use of EF that affects multiple stages of the tick life cycle may also reduce the likelihood of ticks developing resistance [64]. When ticks are targeted at multiple developmental stages, it increases the overall effectiveness of the fungal agents, as ticks have less opportunity to adapt or survive through one specific stage.

In our study, the most effective isolates against the tested development stages of *I. ricinus* were two environmental strains of *Metarhizium anisopliae* (3.4(2) collected from the soil in CFs and 6.4(6) collected from the soil in BFs), with LC_50_ values ranging from 1.6 × 10^5^ up to 1.8 × 10^6^ cfu/mL. However, different mortality rates of the different stages of *I. ricinus* were observed. The study found that for most strains, females are the most sensitive developmental stage and most susceptible to EF, while males were more resistant to the strains used. A high mortality rate among adult *I. ricinus* was also noted in a previous study by Szczepanska et al. [37], who found that females are more susceptible to the environmental strain *M. anisopliae* compared to males (LC_50_ 2.6 × 10^3^ cfu/mL for females and 5.2 × 10^4^ cfu/mL for males). A lower mortality rate, similar to that obtained in our study, was reported in a study by Fernández-Salas et al. [61] among *R. microplus* larvae by a strain of *M. anisopliae* (MaV50), for which the LC_50_ value was 1.3 × 10^6^ cfu/mL. Additionally, in our study, nymphs were found to be less susceptible to EF than adults. A previous study by Samish et al. [64] on *R. sanguineus* nymphs treated with *M. anisopliae* showed that the reduced mortality of nymphs compared to adults may be due to differences in the cuticle composition. Higher mortality rates are typically observed in engorged ticks compared to unfed ticks and in adult ticks compared to juvenile ticks. In addition, moving beyond laboratory conditions to field trials would offer invaluable data on the real-world effectiveness of EF in controlling tick populations. These studies can also reveal practical challenges and the impact of environmental variability on EF efficacy. Further, more comprehensive research is required to analyze the impact of the presence of fungi on the tick population.

## 5. Conclusions

Although the microclimate and host availability are important factors in maintaining tick populations, the presence of entomopathogenic fungi in the ecosystem may also influence tick dynamics. This study highlighted the common presence of entomopathogenic fungi in the tick habitat, with a lethal potential against the local tick population. Therefore, the soil collected from the forest habitats of *Ixodes ricinus* can be an important source of entomopathogenic fungi. However, the number of species and isolates of EF, as well as their effectiveness against ticks, varies between sites. The locally sourced entomopathogenic fungi are well adapted to local microclimatic conditions, making them promising candidates for biocontrol. Nevertheless, in order to advance these findings toward practical applications, further laboratory tests and extensive field testing are required to select the best entomopathogenic fungi isolate and to confirm the effectiveness and safety of entomopathogenic fungi in natural habitats.

## Figures and Tables

**Table 1:** Entomopathogenic fungi isolated from soil samples collected from broadleaf forests (BFs), mixed broadleaf and coniferous forests (MBCFs), and coniferous forests (CFs).

Type of Forest Habitat	Site	Number of EF Isolates (%)		
		*Metarhizium*	*Beauveria*	*Isaria*
BF	1	4	4	-
	6	15	-	1
	9	1	-	10
	Total	20 (37.7)	4 (7.5)	11 (20.8)
MBCF	2	12	1	-
	5	-	-	-
	7	-	-	-
	Total	12 (22.7)	1 (1.9)	-
CF	3	2	-	-
	4	1	-	-
	8	2	-	-
	Total	5 (9.4)	-	-
Total		37 (69.8)	5 (9.4)	11 (20.8)

**Table 2:** Median lethal concentration (LC_50_) of different developmental stages of *Ixodes ricinus* infected with different fungal strains of the genera *Metarhizium* and *Beauveria*.

Strain	Germination (%)	Developmental Stage of Ticks		
		LC_50_ (cfu/mL)		
		Females	Males	Nymphs
1.3(3) *Metarhizium* sp.	92	8.5 × 10^5^	3.4 × 10^6^	1.2 × 10^7^
1.4(4) *Beauveria bassiana*	96	1.9 × 10^7^	2.9 × 10^6^	2.6 × 10^6^
2.3(1) *Beauveria* sp.	91	2.9 × 10^6^	3.0 × 10^6^	6.6 × 10^7^
3.4(2) *Metarhizium anisopliae*	95	6.1 × 10^5^	1.6 × 10^5^	1.4 × 10^6^
6.4(6) *Metarhizium anisopliae*	92	2.9 × 10^5^	1.8 × 10^6^	1.9 × 10^6^
9.4(4) *Metarhizium anisopliae*	90	2.8 × 10^6^	1.3 × 10^7^	1.4 × 10^7^

## Data Availability

The data presented in this study are available on request from the first author.
